# Supplementary material for: Multimodal cell atlas of the ageing human skeletal muscle
Source: Nature. 2024 Apr 22;629(8010):154–64. doi: 10.1038/s41586-024-07348-6 (PMC11062927; doi:10.1038/s41586-024-07348-6)
Supplement: Supplementary file 1 — Reporting Summary [file 41586_2024_7348_MOESM1_ESM.pdf]

nature portfolio

Corresponding author(s): Pura Munoz-Canoves and Miguel A. Esteban

Last updated by author(s): Mar 8, 2024

Reporting Summary

Nature Portfolio wishes to improve the reproducibility of the work that we publish. This form provides structure for consistency and transparency in reporting. For further information on Nature Portfolio policies, see our Editorial Policies and the Editorial Policy Checklist.

Statistics

For all statistical analyses, confirm that the following items are present in the figure legend, table legend, main text, or Methods section.

n/a	Confirmed	
☐	☒	The exact sample size (*n*) for each experimental group/condition, given as a discrete number and unit of measurement
☒	☐	A statement on whether measurements were taken from distinct samples or whether the same sample was measured repeatedly
☐	☒	The statistical test(s) used AND whether they are one- or two-sided<br>*Only common tests should be described solely by name; describe more complex techniques in the Methods section.*
☐	☒	A description of all covariates tested
☒	☐	A description of any assumptions or corrections, such as tests of normality and adjustment for multiple comparisons
☐	☒	A full description of the statistical parameters including central tendency (e.g. means) or other basic estimates (e.g. regression coefficient) AND variation (e.g. standard deviation) or associated estimates of uncertainty (e.g. confidence intervals)
☐	☒	For null hypothesis testing, the test statistic (e.g. *F*, *t*, *r*) with confidence intervals, effect sizes, degrees of freedom and *P* value noted<br>*Give P values as exact values whenever suitable.*
☒	☐	For Bayesian analysis, information on the choice of priors and Markov chain Monte Carlo settings
☒	☐	For hierarchical and complex designs, identification of the appropriate level for tests and full reporting of outcomes
☐	☒	Estimates of effect sizes (e.g. Cohen's *d*, Pearson's *r*), indicating how they were calculated

Our web collection on statistics for biologists contains articles on many of the points above.

Software and code

Policy information about availability of computer code

Data collection	DIPSEQ T1 sequencer for droplet-based sn/scRNA-seq. BGISEQ-2000 sequencer for snATAC-seq. Zeiss 980 AiryScan2, Leica DMR6000B or Nikon Ti2 for fluorescence microscope imaging.
Data analysis	Sequencing data were analyzed using: Cutadapt (v1.14), FastQC (v0.11.2), fastp (v0.21.0), STAR (v2.7.4a), PISA (v0.2), sambamba (v0.7.0), Seurat (v4.0.2), Scanpy (v1.8.1), CellChat (v1.1.0), ArchR (v0.9.5), Python (v3.7), DoubletFinder (v2.0.3), FigR (v0.1.0), SoupX (v1.4.8), pheatmap (v1.0.12), FUMA (v1.5.4), Metascape (v3.0), LDSC (v1.0.1), Hotspot (v1.1.1)<br>Imaging data were analyzed using: LAS AF (v4.0), ZenBlue (v3.5), NIS Elements (v4.11.0), Fiji (ImageJ2) (v2.14.0/1.54f).<br>Custom code supporting this study is available at:<br>https://github.com/MGI-tech-bioinformatics/DNBelab_C_Series_HT_scRNA-analysis-software<br>https://github.com/123anjuan/HMA

For manuscripts utilizing custom algorithms or software that are central to the research but not yet described in published literature, software must be made available to editors and reviewers. We strongly encourage code deposition in a community repository (e.g. GitHub). See the Nature Portfolio guidelines for submitting code & software for further information.

Data

Policy information about availability of data

All manuscripts must include a data availability statement. This statement should provide the following information, where applicable:

- Accession codes, unique identifiers, or web links for publicly available datasets
- A description of any restrictions on data availability
- For clinical datasets or third party data, please ensure that the statement adheres to our policy

Raw data can be found at CNGB Nucleotide Sequence Archive (accession code: CNP0004394, CNP0004395, CNP0004494, CNP0004495) and processed data at Human Muscle Ageing Cell Atlas database (https://db.cngb.org/cdcp/hlma/).
Databases used in this study are: CisBP (http://cisbp.ccbr.utoronto.ca/); SNP2TFBS (https://epd.expasy.org/snp2tfbs/); CellChat (http://www.cellchat.org/); GWAS Catalog (https://www.ebi.ac.uk/gwas/).

Research involving human participants, their data, or biological material

Policy information about studies with human participants or human data. See also policy information about sex, gender (identity/presentation), and sexual orientation and race, ethnicity and racism.

Reporting on sex and gender	Sex was determined based on participants self-reporting. Sex was considered as a covariate in statistical analysis. Details are provided in Supplementary Table 1.
Reporting on race, ethnicity, or other socially relevant groupings	Ethnicity or race was not asked to patients, it was determined by the geographic location.
Population characteristics	Participants: 18 patients for the European cohort, and 13 patients for the Chinese cohort. All details are provided in Supplementary Table 1.
Recruitment	Participants were recruited before orthopedic surgery with informed consent. Before obtaining the informed written consent, the medical team provided both verbal and written information about the research study. For the European cohort, participants were informed about the functional state assessment with the Barthel Index and Charlson Index. Details are provided in Supplementary Table 1.
Ethics oversight	The study was performed in accordance with the Declaration of Helsinki. Ethical approval was granted for the European cohort by the Research Ethics Committee of Hospital Arnau de Vilanova (CEIm 28/2019), and for the Chinese cohort by the Institutional Ethics Committee of the First Affiliated Hospital of Guangdong Pharmaceutical University, Guangzhou (China) (2020-ICE-90).

Note that full information on the approval of the study protocol must also be provided in the manuscript.

Field-specific reporting

Please select the one below that is the best fit for your research. If you are not sure, read the appropriate sections before making your selection.

☒ Life sciences ☐ Behavioural & social sciences ☐ Ecological, evolutionary & environmental sciences

For a reference copy of the document with all sections, see nature.com/documents/nr-reporting-summary-flat.pdf

Life sciences study design

All studies must disclose on these points even when the disclosure is negative.

Sample size	Sample size was not predetermined. 22 individuals (387,444 nuclei/cells) were analyzed in this study.
Data exclusions	We used several criteria to filter low-quality cells or nuclei during data analysis: UMI < 1,000, gene < 500, and mitochondria content > 5% in sn/scRNA-seq, and TSS enrichment scores < 5, number of fragments < 1,000 in snATAC-seq.
Replication	The number of individuals used for profiling in replication for each platform is listed below:<br>scRNA-seq: 8 (in total 79,649 cells).<br>snRNA-seq: 22 (in total 212,774 nuclei).<br>snATAC-seq: 17 (in total 95,021 nuclei).<br>For immunostainning or immunohistology, the numbers of replicates are indicated in the corresponding figure legend.
Randomization	Not relevant because there was no group allocation.
Blinding	There was no blinding performed because there was no group allocation.

Reporting for specific materials, systems and methods

We require information from authors about some types of materials, experimental systems and methods used in many studies. Here, indicate whether each material, system or method listed is relevant to your study. If you are not sure if a list item applies to your research, read the appropriate section before selecting a response.

Materials & experimental systems

n/a	Involved in the study
☐	☒ Antibodies
☒	☐ Eukaryotic cell lines
☒	☐ Palaeontology and archaeology
☐	☒ Animals and other organisms
☒	☐ Clinical data
☒	☐ Dual use research of concern
☒	☐ Plants

Methods

n/a	Involved in the study
☒	☐ ChIP-seq
☒	☐ Flow cytometry
☒	☐ MRI-based neuroimaging

Antibodies

Antibodies used

Primary antibodies:
PAX7 (Developmental Studies Hybridoma Bank cat: PAX7, 1:50)
PDGFRa(eBioscience cat: 17-1401-81, 1:100)
Perilipin (Cell Signalling cat: 9349, 1:100)
Filamin C (MyBiosource cat: MBS2026155, 1:100)
TNNT2 (Bioss cat:10648R-A488, 1:100)
CD11b (eBioscience cat: 14-0112-85, 1:100)
CD3 (Invitrogen cat: 14-0038-82, 1:100)
CD19 (Invitrogen cat: 14-0199-82, 1:100)
NCAM1 (Cell Sciences cat: Mon9006-1, 1:100)
MyHC Type I (Developmental Studies Hybridoma Bank cat: A4.840)
MyHC Type II (Developmental Studies Hybridoma Bank cat: SC-71, 1:70)
Laminin-647 (Novus Biologicals cat: NB300-144AF647, 1:200)
cFOS (Cell Signaling cat: #2250S, 1:200)
ACVR2A (R&D cat: AF340, 1:100)
ITGA7 (Biocell Scientific cat: 10007, 1:100)
DYSTROPHIN (Sigma cat: D8168, 1:100)

Secondary antibodies (Invitrogen, Carlsbad, CA, USA) were coupled to Alexa Fluor 488, 568, 594 or 647 fluorochromes are listed below diluted at 1:500:
Goat anti-Mouse IgM Cross-Adsorbed Secondary Antibody, DyLight™ 550 (Invitrogen™ cat: SA5-10151)
Goat Anti-Mouse IgG1 Cross-Adsorbed Secondary Antibody Alexa Fluor 488 (Invitrogen™ cat: A21121)
Goat anti-Mouse IgG (H+L) Cross-Adsorbed Secondary Antibody, Alexa Fluor™ 488 (Invitrogen™ cat: A11001)
Goat anti-Mouse IgG (H+L) Cross-Adsorbed Secondary Antibody, Alexa Fluor™ 568 (Invitrogen™ cat: A11004)
Goat anti-Rabbit IgG (H+L) Highly Cross-Adsorbed Secondary Antibody, Alexa Fluor™ Plus 488 (Invitrogen™ cat: A32731TR)
Goat anti-Rabbit IgG (H+L) Highly Cross-Adsorbed Secondary Antibody, Alexa Fluor™ Plus 647 (Invitrogen™ cat: A32733TR)
Donkey anti-Goat IgG (H+L) Highly Cross-Adsorbed Secondary Antibody, Alexa Fluor™ Plus 647 (Invitrogen™ cat: A32849TR)
Goat anti-Rat IgG (H+L) Cross-Adsorbed Secondary Antibody, Alexa Fluor™ 568 (Invitrogen™ cat: A11077)

Validation

All antibodies are commonly used in the field and have been validated by the manufacturer. For the primary antibodies, the following information were provided by the supplier:
PAX7 (Developmental Studies Hybridoma Bank cat: PAX7, 1:50), reacts with: Amphibian, Avian, Axolotl, Bovine, Canine, Fish, Goat, Human, Mouse, Ovine, Porcine, Quail, Rat, Turtle, Xenopus, Zebrafish; suitable for; Chromatin Immunoprecipitation, FACS, FFPE, Gel Supershift, Immunofluorescence, Immunohistochemistry, Immunoprecipitation, Western Blot; validated in adult skeletal muscle satellite cells.
PDGFRa(eBioscience cat: 17-1401-81, 1:100), reacts with: Human, Mouse; suitable for IHC, ICC/IF, Flow cytometry; validated in NIH/3T3 cells.
Perilipin (Cell Signalling cat: 9349, 1:100), reacts with: Human, Mouse; suitable for Western Blot, Immunoprecipitation, Immunohistochemistry, Chromatin Immunoprecipitation, CUT&RUN, CUT&Tag, Dot Blot, eCLIP, Immunofluorescence, Flow Cytometry; validated in adipocyte.
Filamin C (MyBiosource cat: MBS2026155, 1:100), reacts with: Human; suitable for Western Blot, Immunohistochemistry; validated in Human breast cancer tissue, Human prostate tissue, Human colorectal cancer tissue, Human prostate tissue.
TNNT2 (Bioss cat:10648R-A488, 1:100), reacts with: Human, Mouse, Rat; suitable for WB, FCM, IF(IHC-P), IF(IHC-F), IF(ICC); validated in cardiac muscle.
CD11b (eBioscience cat: 14-0112-85, 1:100), reacts with Rabbit, Bat, Fish, Mouse, Human; suitable for Western Blot, Immunohistochemistry, Immunocytochemistry, Immunoprecipitation, Flow Cytometry; validated in mouse splenocytes.
CD3 (Invitrogen cat: 14-0038-82, 1:100), reacts with: Rat, Human, Mouse; suitable for Western Blot, Immunohistochemistry, Immunocytochemistry, Immunoprecipitation, Flow Cytometry; validated in lymphocytes.
CD19 (Invitrogen cat: 14-0199-82, 1:100), reacts with: Human; suitable for Immunohistochemistry, Flow Cytometry; validated in B-lymphocyte.

NCAM1 (Cell Sciences cat: Mon9006-1, 1:100), reacts with: Human; suitable for IHC-F, IHC-P; validated in small cell carcinomas and carcinoids of the lung.
MyHC Type I (Developmental Studies Hybridoma Bank cat: A4.840), reacts with: Rat, Human, Mouse; suitable for IHC, WB, IF, ICC; validated in skeletal muscle.
MyHC Type II (Developmental Studies Hybridoma Bank cat: SC-71, 1:70), reacts with: Rat, Human, Mouse; suitable for IHC, WB, IF, ICC; validated in skeletal muscle.
Laminin-647 (Novus Biologicals cat: NB300-144AF647, 1:200), reacts with: Rat, Human, Mouse, Rabbit, Fruit Bat, Chinese Hamster; suitable for WB, Flow, ICC/IF, IHC, IHC; validated in epithelial tissue, nerve, fat cells and smooth, striated and cardiac muscle.
cFOS (Cell Signaling cat: #2250S, 1:200), reacts with: Human, Mouse, Rat; suitable for Western Blot, Immunoprecipitation, Immunohistochemistry, Chromatin, Immunoprecipitation, CUT&RUN, CUT&Tag, eCLIP, Immunofluorescence, Flow Cytometry; validated in fibroblasts.
ACVR2A (R&D cat: AF340, 1:100), reacts with: Human; suitable for Western Blot, Immunohistochemistry; validated in Human prostate cancer tissue.
ITGA7 (Biocell Scientific cat: 10007, 1:100), reacts with: Human, Mouse, Rat; suitable for Western Blot, Immunohistochemistry; validated in rodent and human tissues.
DYSTROPHIN (Sigma cat: D8168, 1:100), reacts with: Chicken, Rat, Human, Pig, Rabbit, Mouse; suitable for Western Blot, Immunohistochemistry; validated in mouse muscle.

Animals and other research organisms

Policy information about studies involving animals; ARRIVE guidelines recommended for reporting animal research, and Sex and Gender in Research

Laboratory animals	C57Bl/6 (WT) mice were bred and raised till 8-12 weeks of age at the animal facility of the Barcelona Biomedical Research Park (PRBB). They were housed in standard cages with a 12-hour light-dark cycle and given unrestricted access to a standard chow diet.
Wild animals	N/A
Reporting on sex	Both males and females were used for experiments and were maintained according to the Jackson Laboratories's guidelines and protocols.
Field-collected samples	N/A
Ethics oversight	All experiments adhered to the 'three Rs' principle—replacement, reduction, and refinement—outlined in Directive 63/2010 and its implementation in Member States. Procedures were approved by the PRBB Animal Research Ethics Committee (PRBB-CEEA) and the local government (Generalitat de Catalunya), following European Directive 2010/63/EU and Spanish regulations RD 53/2013.

Note that full information on the approval of the study protocol must also be provided in the manuscript.
